# Genomic alterations and dynamic molecular residual disease monitoring predict pathological response to neoadjuvant chemoimmunotherapy in esophageal squamous cell carcinoma

**DOI:** 10.3389/fimmu.2026.1681959

**Published:** 2026-04-30

**Authors:** Dijian Shen, Jinxiao Liang, Junrong Yan, Weishan Lu, Da Chen, Kaiyi Tao, Mengyun Wang, Sheng Chen, Qixun Chen

**Affiliations:** 1 Department of Thoracic Oncology Surgery, Zhejiang Cancer Hospital, Hangzhou Institute of Medicine, Chinese Academy of Sciences, Hangzhou, China; 2 Medical Department, Nanjing Geneseeq Technology Inc., Nanjing, China

**Keywords:** esophageal squamous cell carcinoma (ESCC), major pathological response (MPR), molecular residual disease (MRD), neoadjuvant chemoimmunotherapy (NCIT), *NOTCH1*

## Abstract

**Background:**

Approximately 40-60% of patients with locally advanced esophageal squamous cell carcinoma (ESCC) exhibit suboptimal responses to neoadjuvant chemoimmunotherapy (NCIT), highlighting the need for predictive biomarkers of pathological response.

**Methods:**

We prospectively enrolled 29 stage II-III ESCC patients receiving NCIT (albumin-paclitaxel/carboplatin + anti-PD-1). Baseline tumor tissues were firstly analyzed via 437-gene targeted sequencing. Serial preoperative plasma samples collected before NCIT, during NCIT and post-NCIT/pre-surgery, along with baseline tissue, were profiled using a 2365-gene panel for tumor-informed molecular residual disease (MRD) monitoring. Associations between clinicopathological features, genomic alterations, MRD status, and major pathological response (MPR) were evaluated.

**Results:**

No clinicopathological feature significantly correlated with MPR. MPR was significantly associated with baseline *NOTCH1* mutations (*p* = 0.014) and higher chromosomal instability score (CIS, *p* = 0.032). Absence of MRD and lower ctDNA levels after NCIT completion, but not before and during, strongly correlated with MPR. Distinct change patterns of ctDNA level from on- to post-NCIT were observed in MPR versus non-MPR patients. A model integrating baseline *NOTCH1* mutation and post-NCIT MRD status achieved superior MPR prediction (AUC = 0.954), outperforming either factor alone (*NOTCH1*, AUC = 0.769; post-NCIT MRD, AUC = 0.882).

**Conclusions:**

Dynamic MRD monitoring, particularly post-treatment, provides strong predictive value for pathological response to NCIT in locally advanced ESCC. Integrating baseline *NOTCH1* mutation status with post-NCIT MRD assessment significantly improves MPR prediction, indicating the potential to inform clinical decision-making for these patients.

## Background

Esophageal cancer represents a critical health burden in China, accounting for 43.8% of global incidence and 42.1% of mortality in 2022, respectively (1). Esophageal squamous cell carcinoma (ESCC) constitutes the predominant histologic subtype in this population (2). Due to its insidious onset and aggressive propensity for early lymph node metastasis, the majority of patients are diagnosed at advanced stages, contributing to persistently poor outcomes. The reported 5-year survival rate for ESCC remains limited to approximately 15-20% (3), highlighting the critical need for therapeutic advances to improve prognosis.

For early-stage ESCC (cT1-2N0M0), radical esophagectomy remains standard treatment option (4). However, in locally advanced resectable ESCC, current guidelines recommend neoadjuvant chemoradiotherapy (NCRT) or chemoimmunotherapy (NCIT) followed by surgery (4). Currently, numerous clinical studies investigating neoadjuvant chemotherapy combined with programmed death‐1 (PD-1) or programmed death ligand 1 (PD-L1) inhibitors for ESCC have yielded remarkable outcomes, establishing NCIT as the preferred treatment regimen for ESCC (5, 6). Nevertheless, approximately 40-60% of patients exhibit suboptimal responses, highlighting the critical need for biomarkers predicting NCIT efficacy to guide therapeutic decisions.

Molecular residual disease (MRD) represents the presence of trace amounts of cancer through liquid biopsy following treatment. Circulating tumor DNA (ctDNA)-based MRD detection represents a highly sensitive method for predicting recurrence risk and therapeutic outcomes (7). This approach shows significant promise in guiding treatment decisions across neoadjuvant and adjuvant settings, facilitating optimized clinical management (7). Yue et al. demonstrated that MRD profiling effectively identifies ESCC patients achieving pathological complete response (pCR) post-surgery and those likely to remain progression-free without surgical intervention (8). However, comprehensive analyses correlating MRD status and dynamic changes in MRD with pathological outcomes in NCIT for ESCC remain limited, highlighting a critical research gap.

In this study, we performed genomic profiling of pre-treatment tumor tissue and serial liquid biopsies in locally advanced ESCC patients undergoing NCIT. Plasma samples were collected at three critical timepoints: before treatment (baseline, P1), during treatment (on-NCIT, P2), and after treatment completion (post-NCIT, P3). We implemented tumor-informed ctDNA monitoring using a customized 2365-gene panel, with MRD status and ctDNA levels rigorously correlated with major pathological response (MPR) at surgery. This study aimed to assess the predictive value of baseline genomic features, MRD status and dynamic changes of ctDNA levels during NCIT for predicting pathological outcomes.

## Materials and methods

### Study design and participants

A total of 29 patients with ESCC who received neoadjuvant chemotherapy combined with PD-1 inhibitor at Zhejiang Cancer Hospital between Mar 2023 and Oct 2024 were included. Clinical staging of primary tumors was done according to the American Joint Committee on Cancer guidelines (AJCC, 8th edition). All ESCC patients received 2–4 cycles of treatment with the regimen consisting of albumin-bound paclitaxel + carboplatin/nedaplatin + PD-1 inhibitor. The primary inclusion criteria were as follows: (1) Histologically or cytologically confirmed ESCC. (2) Presence of measurable lesions according to RECIST criteria. (3) Clinical stage cT2-4aNanyM0 or T1-3N+M0 based on the AJCC/UICC esophageal cancer staging (8th edition). (4) Age ≥ 18 years and ≤ 75 years. (5) ECOG performance status score of 0-1. (6) No esophageal perforation, active esophageal bleeding, or obvious invasion of the trachea or major thoracic blood vessels. (7) No prior history of chest chemotherapy, radiotherapy, immunotherapy, or biological therapy. (8) Hemoglobin ≥ 90 g/L, platelets ≥ 10×10^9^/L, absolute neutrophil count ≥ 1.5×10^9^/L. (9) Serum creatinine ≤ 1.25 times the upper normal limit (UNL) or creatinine clearance ≥ 60 mL/min. (10) Serum bilirubin ≤ 1.5 times UNL; Aspartate aminotransferase (AST) and Alanine aminotransferase (ALT) ≤ 2.5 times UNL; alkaline phosphatase ≤ 5 times UNL. (11) No interstitial pneumonia or history of prior interstitial pneumonia. (12) Forced expiratory volume in 1 second (FEV1) > 1.2 L. (13) Signed formal informed consent by the patient. Exclusion criteria included: (1) Having received chest radiotherapy, chemotherapy, immunotherapy or surgical resection for esophageal cancer before the start of this trial. (2) Cervical esophageal cancer. (3) Esophageal perforation, active esophageal bleeding, or invasion of the trachea or major thoracic blood vessels. (4) Patients with severe cardiovascular or pulmonary diseases, interstitial pneumonia, or a history of prior interstitial pneumonia. (5) Patients with obvious esophageal ulcers, moderate or severe chest/back pain, or symptoms of esophageal perforation. (6) Patients who cannot understand the trial requirements or are likely to fail to comply with them. (7) Presence of hematogenous metastasis. (8) Autoimmune diseases (e.g., systemic lupus erythematosus, rheumatoid arthritis, inflammatory bowel disease, autoimmune thyroid disease), except for the following diseases which are allowed to proceed to the next stage of screening: type 1 diabetes, skin diseases that do not require systemic treatment (e.g., vitiligo, psoriasis). (9) Active hepatitis B or hepatitis C requiring treatment. (10) Having an active infection requiring systemic treatment within 14 days before the first dose administration. (11) Patients with other malignant lesions, except for curable skin cancers (non-melanoma), carcinoma *in situ* of the cervix, or malignant diseases that have been cured for ≥ 5 years. (12) Obvious diseases that the investigator deems should be excluded from this study. This study was performed in accordance with the Declaration of Helsinki and the Good Clinical Practice guidelines. All protocols were approved by the Ethics Committee of Zhejiang Cancer Hospital (approval number: IRB-2022-498), and written informed consent was obtained from each participant before sample collection.

### Pathological assessments

Pathological response was independently determined by two pathologists. Any disagreements were resolved by consensus. Patients achieving major pathological response (MPR) were designated MPR group, while those did not achieve MPR constituted non-MPR group for subsequent molecular biomarker analyses.

### Samples collection and preparation

Pre-treatment tumor samples were obtained by endoscopic biopsy and subjected to genomic alterations analysis. Serial peripheral samples were collected at three time points for ctDNA profiling by targeted next-generation sequencing (NGS): baseline (P1, before NCIT initiation), on-NCIT (P2, on cycle 2, day 1 [C2D1] prior to agents administration), and post-treatment (P3, approximately two weeks after NCIT completion). Tumor tissues were prepared into formalin-fixed and paraffin-embedded (FFPE) blocks. For each time point, about 8–10 mL of peripheral blood was collected from each patient in EDTA-coated tubes (BD Biosciences), and plasma was separated within 2 hours after blood collection. While the white blood cells (WBCs) after plasma preparation were also collected and sequenced as normal controls to identify germline mutations and mutations due to clonal hematopoiesis. All samples were sent to a centralized testing center of Nanjing Geneseeq Technology Inc. (Nanjing, China) for targeted NGS.

### DNA extraction, library preparation, and targeted enrichment

Genomic DNA from the white blood cells were extracted using the DNeasy Blood & Tissue Kit (Qiagen). Genomic DNA from FFPE sections was extracted with QIAamp DNA FFPE Tissue kit (Qiagen). Circulating cell-free DNA (cfDNA) from plasma was extracted using the QIAamp Circulating Nucleic Acid kit (Qiagen). DNA was quantified by Qubit 3.0 using the dsDNA HS Assay Kit (Life Technologies), and the quality was evaluated by a Nanodrop 2000 (Thermo Fisher). Genomic DNA was sheared into fragments (300~350 bp) using a Covaris M220 instrument. cfDNA or fragmented genomic DNA underwent sequencing library preparation using KAPA Hyper Prep kit (KAPA Biosystems). Briefly, DNA underwent end repairing, A-tailing, and adaptor ligation and then was amplified by polymerase chain reaction (PCR) and purified. Next, for tissue-derived DNA and baseline plasma-derived cfDNA, hybridization-based target enrichment was carried out using xGen lockdown probes targeting 437 pan-cancer genes (GeneseeqPrime^®^, Nanjing Geneseeq Technology Inc., Nanjing, China), as previously described (9). For all cfDNA samples, customized xGen lockdown probes (Integrated DNA Technologies) targeting the 2,365 cancer-relevant genes (Shielding™ Ultra panel, Nanjing Geneseeq Technology Inc.) were utilized for hybridization capture enrichment. Furthermore, to adhere strictly to the tumor-informed approach for MRD monitoring, we performed targeted capture on the baseline tissue samples using the Shielding™ Ultra Panel containing 2365 genes. The enriched libraries underwent on-beads PCR amplification and purification.

### Library sequencing and bioinformatics analysis

The target-enriched library was then sequenced on BGI DNBSEQ-T7 platform according to the manufacturer’s instructions. The mean coverage sequencing depth set when throughput arrangement was 1500× and 6000× for the tumor tissues and WBCs samples, respectively. For cfDNA samples, the mean depth set was 30000×. Mutation calling was performed according to previous study (10). Briefly, trimmomatic was used for FASTQ file quality control, leading/trailing low quality or N bases were removed. Qualified reads were then mapped to reference human genome (hg19) using Burrows-Wheeler Aligner. PCR duplicates were removed by Picard (Broad Institute, MA, USA) after local realignment around known indels and base quality recalibration using Genome Analysis Toolkit (GATK 3.4.0). Matched WBCs control samples were used to filter out germline variants. For tissue samples, somatic single nucleotide variants (SNV) and insertion/deletions (indels) were detected using VarScan2, with minimum variant allele frequency threshold set was ≥ 1%, and a minimum of six unique mutant reads on different strands with good quality scores.

For cfDNA samples, sequencing data were processed as previously described (10). Given that tumor-associated mutations have been identified in the physiologically normal mucosa of esophageal cancer patients in prior studies (11), and recognizing that such mutations may also be released into peripheral blood, we rigorously applied a tumor-informed screening strategy to cfDNA-derived mutations. Specifically, we focused our plasma screening exclusively on mutations also detected in the baseline tumor tissue sample. Candidate variants were validated if they met the following criteria: i) SNVs/indels with variant allele frequency > 0.02% and supported by at least three high-quality sequencing reads; ii) absence in our published database of clonal hematopoiesis variants (12); iii) not present in the paired WBCs control sample. ctDNA or MRD positivity was defined as the presence of one or more mutations identified in the corresponding cfDNA sample.

All SNVs/indels were annotated with ANNOVAR, and each SNV/indel was manually checked on the Integrative Genomics Viewer (IGV). Gene fusions were identified by FACTERA and copy number variations (CNVs) were analyzed with ADTEx, with default parameters. For both tissue and cfDNA samples, copy number gain and loss were defined based on log_2_ ratio thresholds of ≥ 2.0 and ≤ -0.6, respectively. Tumor mutational burden (TMB) was defined as the total number of SNVs/indels across the coding region of targeted genes in tumor samples, including synonymous alterations but excluding known driver mutations (13). Microsatellite instability (MSI) status was determined as a tissue sample exhibiting instability at more than 40% of 52 indel sites, relative to control samples (14). The mean percentage of genes with abnormal (log2 depth ratio > ± 0.2) copy numbers, weighted on 22 autosomal chromosomes, was defined as chromosomal instability score (CIS). Intratumor heterogeneity (ITH) of tumors was quantified via PyClone (v0.13.0) as previously described (15). The ctDNA concentration was classified as haploid genome equivalents (hGE) per mL of plasma (hGE/mL) and calculated with the following formula: [(the mean VAF for all mutations detected) × cfDNA concentration×1000 (ng/mL of plasma)] ÷ 3.3, as previously described (16).

### Statistical analysis

Quantitative data were presented as median (range) or number of patients (percentage). Fisher’s exact test assessed differences in categorical variables across groups, while the Wilcoxon rank-sum test was used to compare the distribution of continuous data. For diagnostic modeling, a multivariable logistic regression model was fitted to predict the probability of major pathological response (MPR), and a nomogram was constructed to visualize the model using the rms package. Model discrimination was evaluated by the area under the curve (AUC) of the receiver operating characteristic (ROC). Model calibration was assessed via 1,000-iteration bootstrap resampling to derive the bias-corrected calibration slope, intercept, mean absolute error (MAE) and Brier score. The goodness-of-fit was further evaluated using the Hosmer-Lemeshow test. To ensure model robustness and address the potential risk of overfitting, internal validation was performed using leave-one-out cross-validation (LOOCV). The cross-validated AUC and its 95% confidence interval (CI) were estimated through 1,000-iteration bootstrap resampling of the LOOCV outcomes. Unless otherwise specified, all quoted P-values were two-sided, with those less than 0.05 considered statistically significant. All statistical analyses were performed in R (version 4.1.3).

## Results

### Patient characteristics and sample collection for NGS

A total of 29 patients with TNM stage II–III ESCC were included in this study. All patients received neoadjuvant chemoimmunotherapy consisting of albumin-bound paclitaxel plus carboplatin or nedaplatin in combination with a PD-1 inhibitor. Detailed demographic and clinical characteristics of the cohort are summarized in **Table 1**. The cohort comprised 27 males (93.1%) and had a median age of 69 years (range, 53-75). History of smoking and alcohol consumption was reported in 17 (58.6%) and 18 patients (62.1%), respectively. Tumor distribution across esophageal subsites included the upper esophagus in two patients (6.9%), middle esophagus in 14 (48.3%), and lower esophagus in 13 (44.8%). Histopathological grading revealed four G1 (13.8%), 15 G2 (51.7%), and nine G3 (31.0%) tumors, with one case (3.4%) unknown. According to AJCC staging criteria (8^th^ version), four patients (13.8%) were classified as stage II and 25 (86.2%) as stage III. Clinical staging demonstrated that 28 patients (96.6%) presented with cT3 tumors, while only one individual (3.4%) exhibited a cT2 lesion. Assessment of lymph node involvement revealed that 20 patients (69.0%) were classified as cN1, five patients (17.2%) as cN2, and four patients (13.8%) showed no evidence of regional lymph node metastasis (cN0). PD-L1 expression analysis showed that 13 cases (44.8%) had a combined positive score (CPS) < 10 or a tumor proportion score (TPS) < 1%, 15 (51.7%) demonstrated a CPS ≥ 10 or TPS ≥ 1%, and unknown in one patient (3.4%).

**Table 1:** Baseline clinicopathological characteristics of ESCC patients included in this study.

Characteristics n (%)	Total n=29	MPR n=9	Non-MPR n=20	p value
Age				0.234
≥ 65y	12 (41.4%)	2 (22.2%)	10 (50.0%)	
≥ 65y	17 (58.6%)	7 (77.8%)	10 (50.0%)	
Sex				0.532
Male	27 (93.1%)	8 (88.9%)	19 (95.0%)	
Female	2 (6.9%)	1 (11.1%)	1 (5.0%)	
Smoking history				0.106
Yes	17 (58.6%)	3 (33.3%)	14 (70.0%)	
No	12 (41.4%)	6 (66.7%)	6 (30.0%)	
Alcohol history				0.694
Yes	18 (62.1%)	5 (55.6%)	13 (65.0%)	
No	11 (37.9%)	4 (44.4%)	7 (35.0%)	
Tumor location				0.133
Upper	2 (6.9%)	2 (22.2%)	2(10.0%)	
Middle	14 (48.3%)	6 (66.7%)	8 (40.0%)	
Lower	13 (44.8%)	1 (11.1%)	10 (50.0%)	
Tumor grade				0.734
G1	4 (13.8%)	2 (22.2%)	2 (10.0%)	
G2	15 (51.7%)	4 (44.4%)	11 (55.0%)	
G3	9 (31.0%)	3 (33.3%)	6 (30.0%)	
Unknown	1 (3.4%)	0 (0.0%)	1 (5.0%)	
cT stage				1.000
2	1 (3.4%)	0 (0.0%)	1 (5.0%)	
3	28 (96.6%)	9 (100.0%)	19 (95.0%)	
cN stage				0.779
0	4 (13.8%)	1 (11.1%)	3 (15.0%)	
1	20 (69.0%)	7 (77.8%)	13 (65.0%)	
2	5 (17.2%)	1 (11.1%)	4 (20.0%)	
Clinical stage				1.000
II	4 (13.8%)	1 (11.1%)	3 (15.0%)	
III	25 (86.2%)	8 (88.9%)	17 (85.0%)	
CPS				1.000
<10	13 (44.8%)	4 (44.4%)	9 (45.0%)	
≥10	15 (51.7%)	5 (55.6%)	10 (50.0%)	
Unknown	1 (3.4%)	0 (0.0%)	1 (5.0%)	
TPS (%)				0.435
<1	13 (44.8%)	3 (33.3%)	10 (50.0%)	
≥1	15 (51.7%)	6 (66.7%)	9 (45.0%)	
Unknown	1 (3.4%)	0 (0.0%)	1 (5.0%)	

ESCC, esophageal squamous cell carcinoma; MPR, major pathologic responses; CPS, combined positive score; TPS, tumor proportion score.

**Figure 1:**
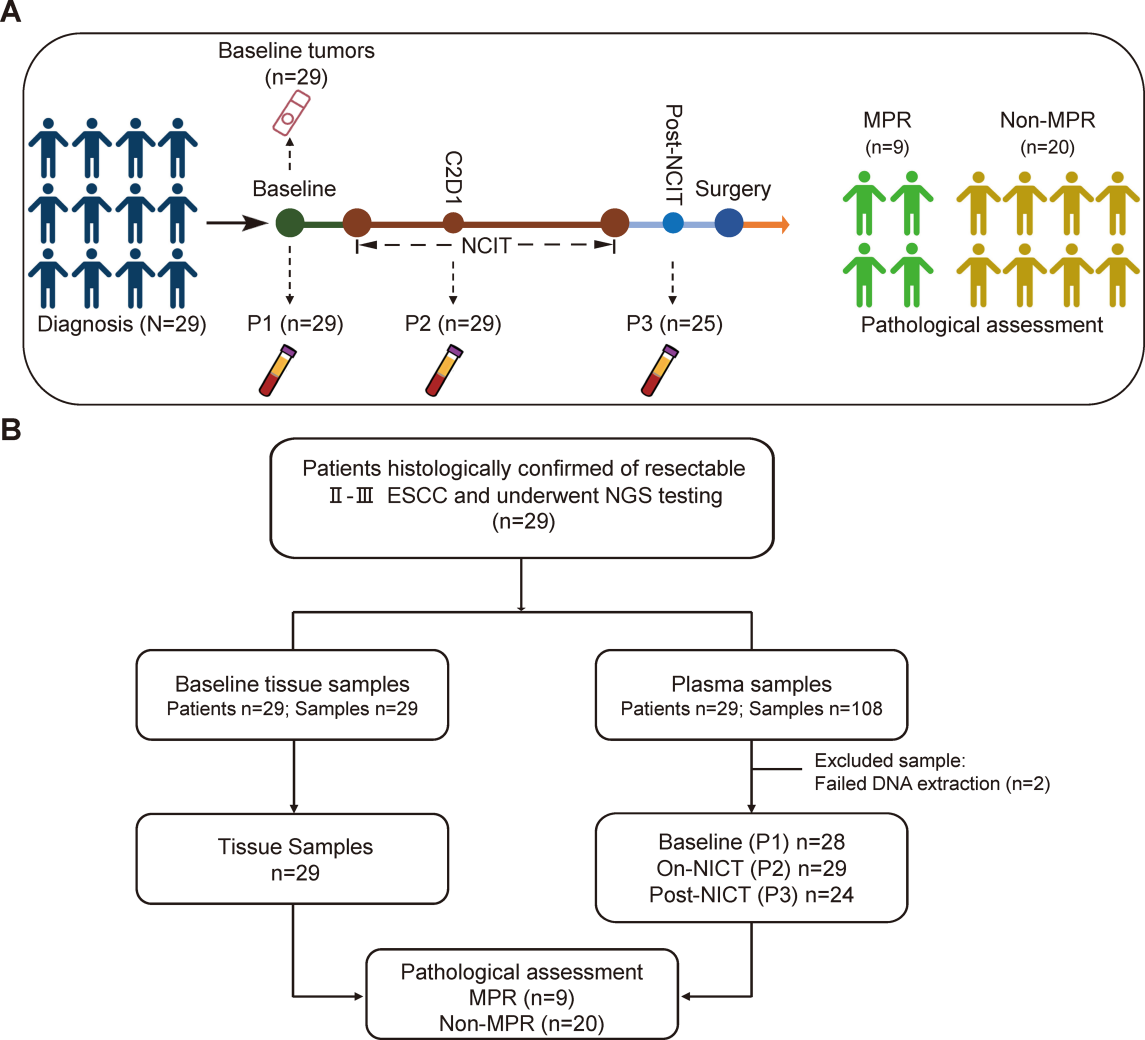
Patient enrollment flowchart. **(A)** Overview of Sample collection and pathological response stratification. **(B)** Flow diagram of patient inclusion and evaluable population. ESCC, esophageal squamous cell carcinoma; NCIT, neoadjuvant chemoimmunotherapy; MPR, major pathological response; C2D1, cycle 2 day 1.

As illustrated in **Figure 1A**, we enrolled 29 patients diagnosed with stage II-III ESCC who had not received any prior treatment before initiating NCIT. After obtaining informed consent, pre-treatment endoscopic biopsy tissues and plasma samples (P1) were collected from all participants for NGS testing, with one plasma sample failed in DNA extraction. For MRD monitoring, longitudinal blood samples were collected: on-treatment samples (P2) were obtained from all 29 patients during NCIT (on-NCIT), while post-NCIT samples (P3) were collected one week after treatment completion from 25 patients (with one plasma extraction failure (**Figure 1B**).

### Pathological efficacy

All 29 patients underwent esophagectomy following neoadjuvant chemoimmunotherapy (NCIT), achieving R0 resection in all cases. Three patients (10.3%) achieved pCR, and a total of nine patients (31.0%) reached MPR, including the three pCR patients. The remaining 20 patients (69.0%) were classified as non-MPR. Based on these criteria, patients were stratified into an MPR group (n = 9) and a non-MPR group (n = 20) for subsequent analyses (**Table 1**).

### The correlation between clinicopathological characteristics and pathological response

We explored the association between pathological response and various clinical characteristics. Univariate Cox regression analysis indicated that clinicopathological factors such as age, sex, smoking history, alcohol history, tumor location, tumor grade, and clinical TNM stage did not significantly related to the pathological efficacy of NCIT in these ESCC patients (**Table 1**). Although a higher proportion of smokers was observed in non-MPR group, this trend failed to reach statistical significance. Additionally, the PD-L1 expression level evaluated based on either CPS or TPS showed no significant correlation with pathological efficacy (**Table 1**).

### Correlation analysis of molecular characteristics in baseline tissue and pathological response

**Figure 2:**
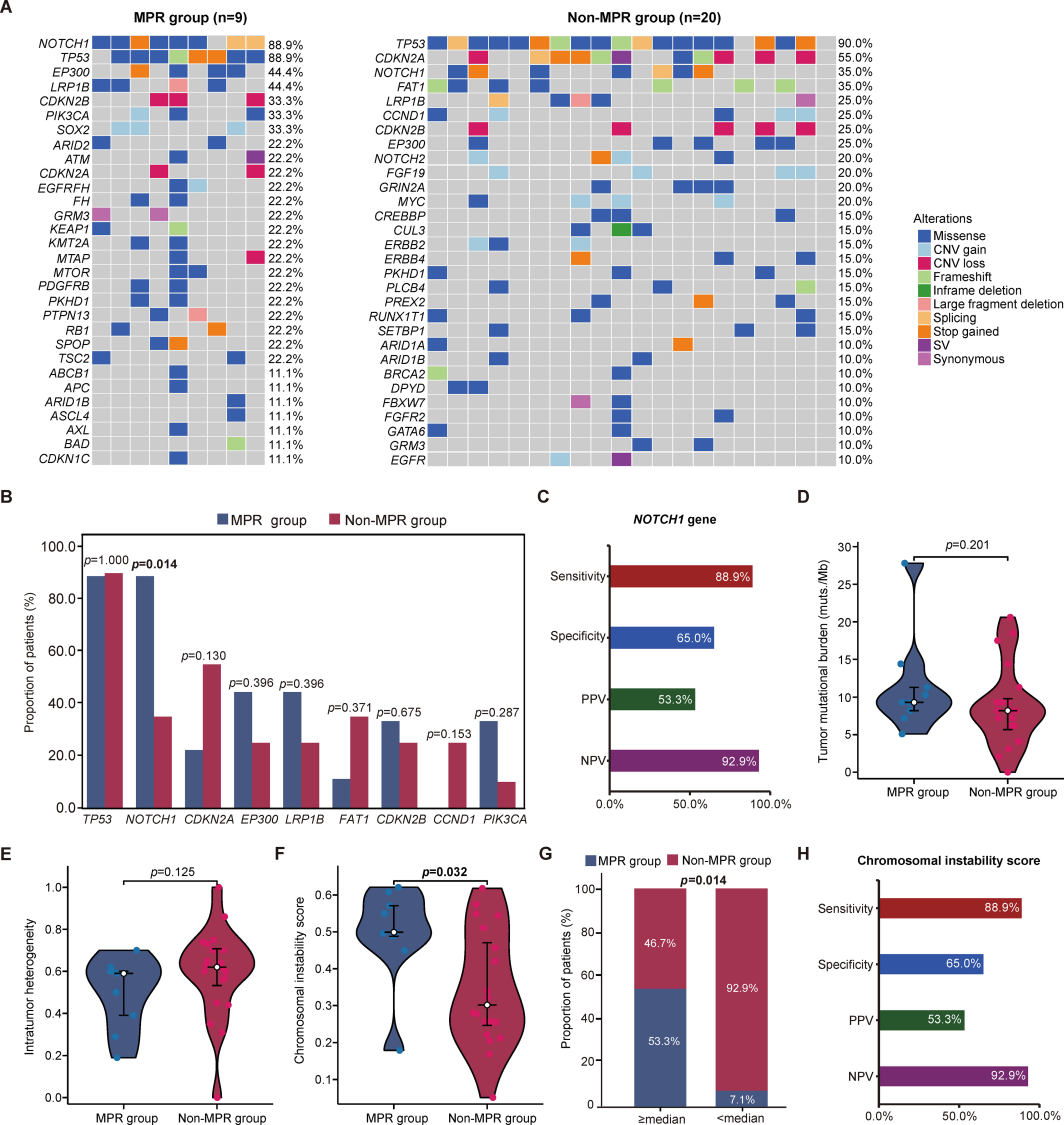
Baseline genomic features of ESCC patients undergoing neoadjuvant chemoimmunotherapy. **(A)** Somatic genomic alterations identified through targeted next-generation sequencing in MPR and non-MPR groups. **(B)** Comparison of gene variant frequencies detected in tumor samples between MPR and non-MPR groups, with statistical significance assessed by Fisher’s exact test. **(C)** The predictive performance of *NOTCH1* mutation status for MPR. **(D–F)** Analysis of tumor mutational burden, intratumoral heterogeneity, and chromosomal instability scores between MPR and non-MPR groups using the Wilcoxon rank-sum test. **(G)** MPR rates stratified by chromosomal instability scores, based on the median cutoff value. **(H)** Evaluation of MPR prediction performance using chromosomal instability scores with median partitioning. MPR, major pathologic responses; CNV, copy number variations; SV, structural variations; CIS, chromosomal instability scores; PPV, positive predictive value; NPV, negative predictive value.

All tissue samples were microsatellite stability (MSS) or low MSI (MSI-L). As shown in **Supplementary Figure S1**, the most commonly altered genes in the 29 patients were *TP53* (89.7%), *NOTCH1* (51.7%), *CDKN2A* (44.8%), *EP300* (31.0%) and *LRP1B* (31.0%). In MPR group, the most frequently altered genes included *NOTCH1* (88.9%), *TP53* (88.9%), *EP300* (44.4%), *LRP1B* (44.4%), *CDKN2B* (33.3%), *PIK3CA* (33.3%) and *SOX2* (33.3%). In non-MPR group, the most frequently altered genes included *TP53* (90.0%), *CDKN2A* (55.0%), *NOTCH1* (35.0%) and *FAT1* (35.0%) (**Figure 2A**). The frequency of *NOTCH1* variants were significantly higher in the MPR subgroup compared to non-MPR group. The detailed profiling of these *NOTCH1* mutations—encompassing nucleotide/amino acid changes, genomic coordinates, variant allele frequency (VAF), and predicted functional impacts—was provided in **Supplementary Table S1**. No other genes demonstrated statistically significant frequency differences between subgroups (**Figure 2B**). We next evaluated the predictive performance of baseline *NOTCH1* mutations for MPR, and found that the detection of baseline *NOTCH1* mutation demonstrated a sensitivity of 88.9% for predicting MPR and a specificity of 35.0% for predicting non-MPR. The positive predictive value (PPV) was 53.3%, while the negative predictive value (NPV) reached 92.9% (**Figure 2C**). Additionally, we analyzed the TMB and found that the median TMB was 8.25 mutations (mut.)/Mb (Range: 2.1-27.8; IQR: 6.95-11.3) in the baseline tissue. The median TMB in MPR and non-MPR group was 9.3 (IQR: 8.2-11.3) and 8.2 (IQR: 5.7-9.8) mut./Mb, respectively. No difference was observed (p = 0.201, **Supplementary Table S2**; **Figure 2D**). Consistently, stratification using a 10 mut./Mb threshold of TMB showed no significant distribution disparity (*p* = 0.396; **Supplementary Table S2**). Furthermore, ITH analysis detected no significant intergroup divergence (*p* = 0.125; **Supplementary Table S2**; **Figure 2E**). Intriguingly, superior pathological response (MPR) was significantly associated with higher CIS (*p* = 0.032; **Supplementary Table S2**; **Figure 2F**). Furthermore, patients were stratified by the median CIS threshold. The proportion achieving MPR was significantly higher among those ≥ the median value compared to those < the median value (*p* = 0.014; **Supplementary Table S2**; **Figure 2G**). Notably, the CIS ≥ median threshold demonstrated predictive performance identical to that of *NOTCH1* mutations, with equivalent metrics: sensitivity 88.9%, specificity 65.0%, PPV 53.3%, and NPV 92.9% (**Figure 2H**).

### Undetectable MRD after completing NICT reliably predicts treatment efficacy

**Figure 3:**
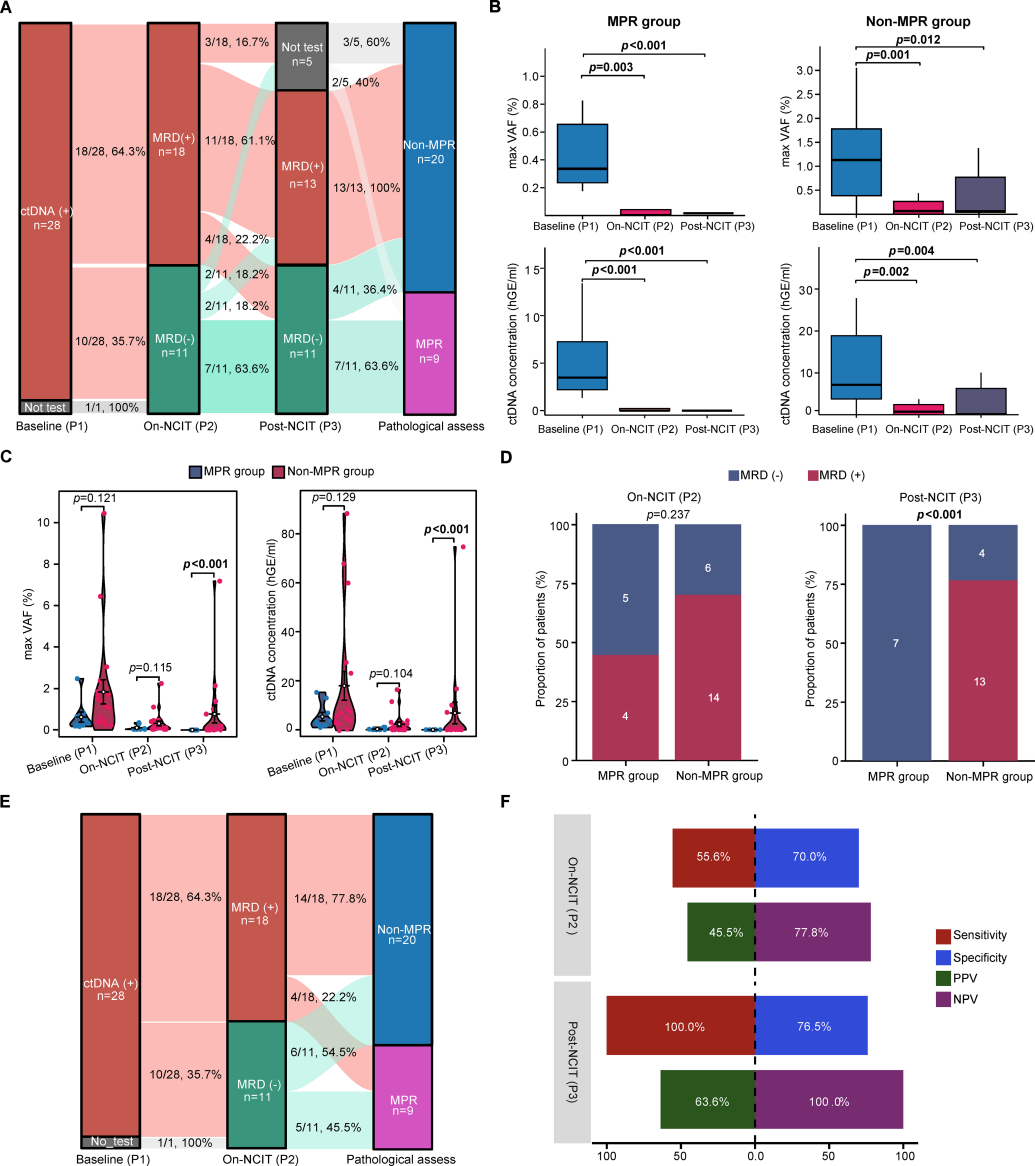
Association between MRD dynamics during NCIT and pathological response. **(A)** Sankey plot illustrating patient distribution stratified by MRD status at baseline, on-NCIT, and post-NCIT. **(B)** Box plots of longitudinal changes in plasma ctDNA metrics: maxVAF and ctDNA concentration. **(C)** Violin plots comparing ctDNA levels (maxVAF and concentration) between MPR and non-MPR groups at three time points. **(D)** MPR/non-MPR rates categorized by MRD status on- and post-NCIT. **(E)** Sankey diagram correlating MRD status on-NCIT with pathological response. **(F)** Predictive performance of undetectable MRD at on- and post-NCIT time points for pathological response assessment. NCIT, neoadjuvant chemoimmunotherapy; MRD, minimal residual disease; MPR, major pathological response; PPV, positive predictive value NPV, negative predictive value.

The feasibility of preoperative plasma ctDNA and the appropriate time in predicting pathological response were evaluated at three time points: at baseline (P1), on-NCIT (P2), and post-NCIT (P3). The positive ctDNA detection rate was 100% (28/28), 62.1% (18/29), and 54.2% (13/24) of P1, P2, and P3, respectively (**Figure 3A**). Analysis of mutation frequencies in baseline plasma-derived alterations revealed no significant difference between MPR and non-MPR groups for any gene variant (*p* > 0.05, **Supplementary Figure S2**). Consequently, baseline ctDNA status has no correlation with the NCIT efficacy and could not predict the pathological response. For patients with matched samples (n = 28) of P1-P2, the P2 MRD detection rates significantly dropped to 64.3% (18/28) (**Figure 3A**), indicating that after one cycle of NCIT administration, some patients achieved molecular clearance in plasma. The P3 MRD positivity rate further declined to 54.2% (13/24), including 11 patients who tested positive in P2 and remained positive in P3, and two patients who were P2 negative but converted to positive in P3. On the contrary, four patients who were P2 positive reverted to negative in P3​ and seven patients who were remained negative from P2 to P3 (**Figure 3A**). All patients with detectable P3 MRD (n = 13) exhibited poorer pathological response (non-MPR). Conversely, in the MRD-negative group (n = 11), seven patients (63.6%) classified as MPR group (**Figure 3A**).

Additionally, the maximum variant allele frequency (maxVAF) and ctDNA concentration quantified as haploid genome equivalents per milliliter (hGE/mL) in P2 and P3 were significantly decreased compared to P1 (*P* < 0.05), demonstrating a reduction in tumor burden during NCIT and after completion of NCIT in both MPR and non-MPR groups (**Figure 3B**). No significant differences in P1 maxVAF (*p* = 0.121) and ctDNA concentration (*p* = 0.129) between MPR and non-MPR groups were observed, indicating that baseline ctDNA levels may not predict the pathological efficacy of NCIT (**Figure 3C**). Although the ctDNA clearance rate was numerically higher in MPR group compared to non-MPR group in P2, this difference did not achieve statistical significance (*p* = 0.237, **Figure 3D**). To further explore whether quantitative ctDNA dynamics during treatment provide predictive information, we analyzed the change from baseline (P1) to P2 and P3 for both maxVAF and ctDNA concentration. At the P2 time point, no significant differences were observed between the MPR and non-MPR groups, whether assessing absolute levels (**Figure 3C**), percent decrease, or log2 fold change (log2 FC) of ctDNA (**Supplementary Figures S3A–D**). However, among patients with available post-NCIT plasma (P3; n = 24), all MPR cases (7/7) achieved ctDNA clearance in P3, whereas only 23.5% (4/17) of non-MPR cases did so. Consequently, the ctDNA clearance rate was significantly higher in MPR group relative to non-MPR group in P3 (*p* < 0.001, **Figure 3D**). Consistently, the MPR group exhibited significantly lower absolute levels of maxVAF and ctDNA concentration (**Figure 3C**) and demonstrated more profound baseline-to-P3 dynamic declines compared to the non-MPR group (**Supplementary Figures S3E–H**; all p < 0.01).

In P3, 100% (13/13) of MRD-positive patients were categorized in non-MPR group, whereas 63.6% (7/11) of ctDNA-negative patients fell within MPR group, with only 36.4% (4/11) demonstrating poorer pathological response (**Figure 3A**). As illustrated in **Figure 3E**, a significant majority of MRD-positive patients in P2 (77.8%, 14/18) had a pathological response of non-MPR, while the remaining 22.2% (4/18) achieved MPR. Among P2 MRD-negative patients, 45.5% (5/11) reached MPR. That is to say, despite achieving MRD negativity in P2, 54.5% (6/11) of patients still exhibited poorer pathological responses. Consequently, the sensitivity of undetectable MRD for predicting MPR was 100% in P3, with a specificity of 76.5% for predicting non-MPR. The PPV was 63.6%, while the NPV was also 100%. These metrics were superior to those in P2, where sensitivity was 55.6%, specificity was 70.0%, PPV was 45.5%, and NPV was 77.8% (**Figure 3F**). In conclusion, the status of plasma MRD after the completion of NCIT (P3) proved to be more effective than that on the therapy (P2) in predicting pathological responses; specifically, the MRD status (positive or negative), the ctDNA level (maxVAF or ctDNA concentration), and the magnitude of ctDNA clearance relative to baseline in P3 showed a significant correlation with pathological efficacy (**Figures 3C, D**; **Supplementary Figures S3E–H**), whereas these metrics at P2, including the dynamic changes from baseline, did not reach statistical significance (**Figure 3D**; **Supplementary Figures S3A–D**).

### Divergent ctDNA dynamics during NCIT between MPR and non-MPR groups

**Figure 4:**
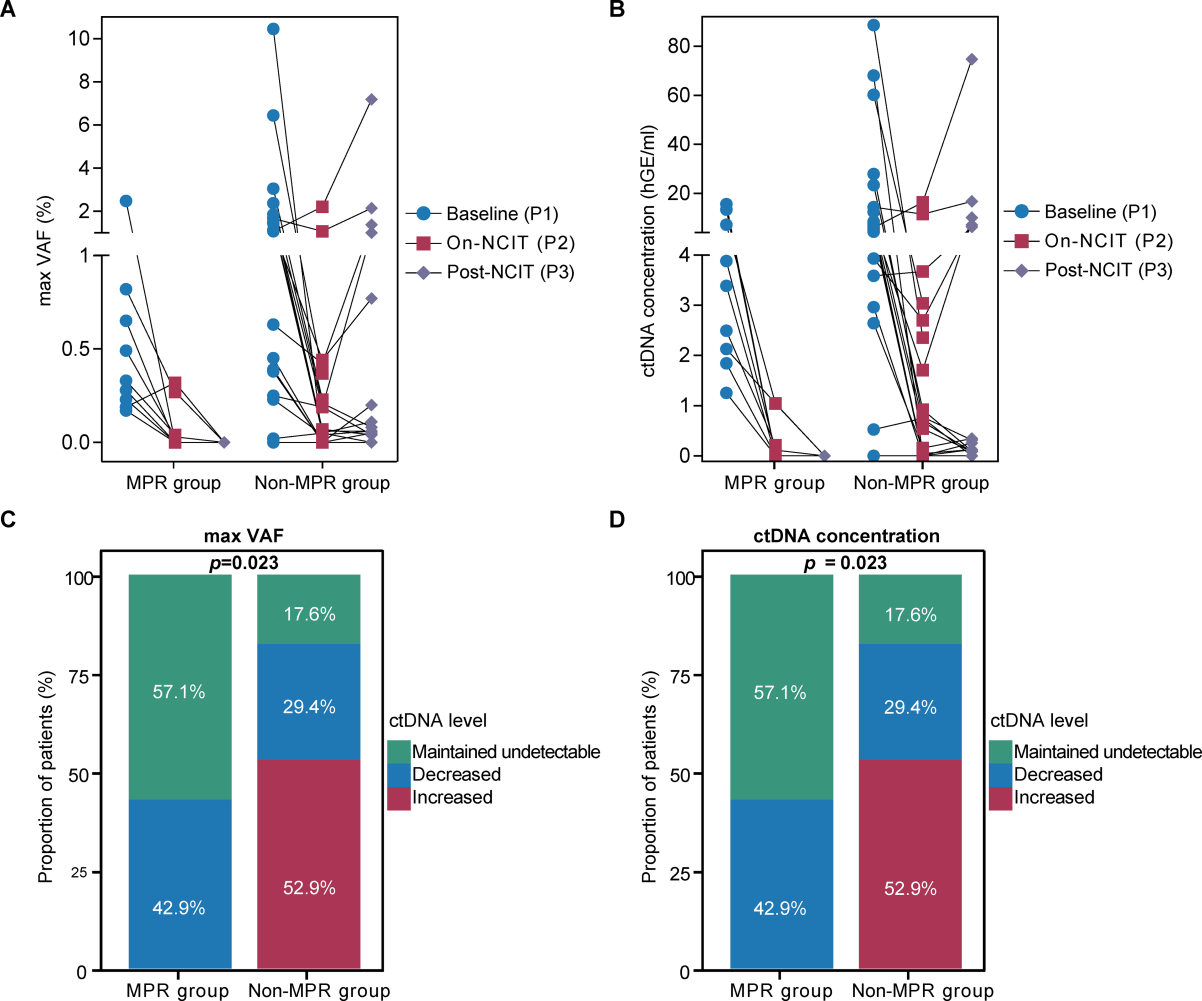
Divergent ctDNA dynamics during NCIT between MPR and non-MPR groups. **(A, B)** Dynamic changes in ctDNA levels at consecutive preoperative time points, including maxVAF and ctDNA concentration. **(C, D)** Distribution of ctDNA dynamics (maxVAF and concentration) from on-NCIT to post-NCIT phases in MPR versus non-MPR groups. NCIT, neoadjuvant chemoimmunotherapy; MPR, major pathological response; maxVAF, the maximum variant allele frequency.

Furthermore, we analyzed the dynamic changes in ctDNA levels at consecutive preoperative time points, including maxVAF and ctDNA concentration. The results showed that in both MPR and non-MPR groups, compared to P1, the maxVAF and ctDNA concentration in P2 decreased (MPR, n = 4; non-MPR, n = 12) or even fell to zero (MPR, n = 5; non-MPR, n = 6) in the majority of patients, except for three patients showed an increase (**Figures 4A, B**). However, at the next time point, after the completion of NCIT treatment, changes in ctDNA levels differed between MPR and non-MPR groups. In MPR group, ctDNA levels either further decreased to zero (3/7) or remained undetectable (4/7). In contrast, in non-MPR group, 52.9% (9/17) of patients experienced an increase (7/9) or conversion from undetectable to detectable (2/9). Three patients remained undetectable, and only five patients showed further decreases (**Figures 4C, D**). Notably, ctDNA concentrations in five patients in non-MPR group were even higher than their baseline values in P3 (**Figure 4B**).

### Prediction model for pathological response to NCIT

**Figure 5:**
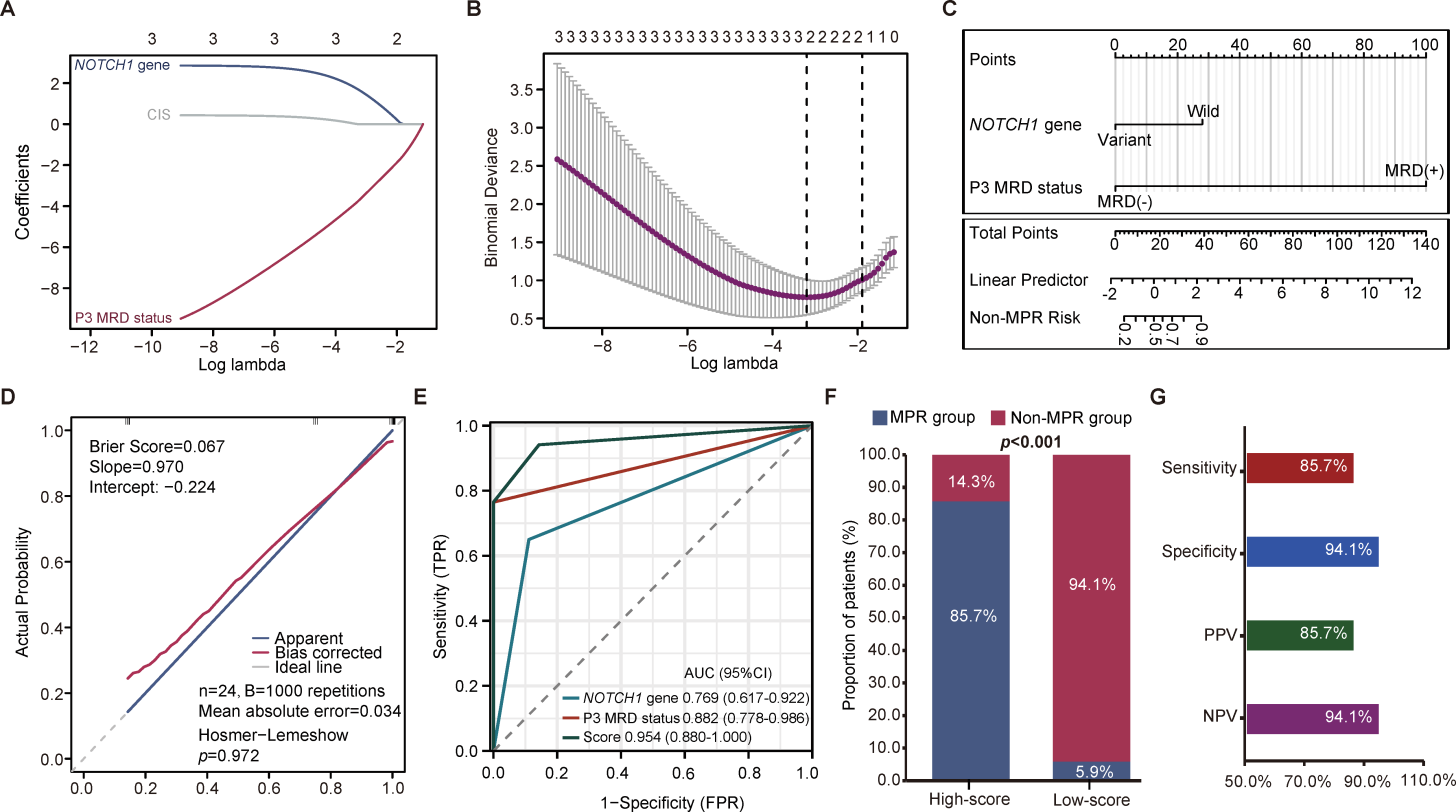
Prediction model for pathological response to NCIT. **(A)** Lasso regression screened the most robust predictors associated with MPR from baseline *NOTCH1* mutation, CIS, and P3 MRD status. **(B)** Cross-validation selected the best lambda for Lasso regression. **(C)** A nomogram was built for estimation of the MPR probability. **(D)** Internal validation using 1000× bootstrap resamples yielded a bias-corrected slope of 0.970 and an intercept of -0.224, and a MAE of 0.038, collectively indicating high predictive accuracy and minimal systemic bias. The Hosmer-Lemeshow test (*p* = 0.972) and Brier score (0.067) further confirmed the model’s excellent goodness-of-fit. **(E)** The model achieved a higher AUC than *NOTCH1* mutation or P3 MRD status alone. **(F)** Stratification of patients into High- and Low-score groups based on the Youden index-derived optimal threshold. The High-score group exhibited significantly higher MPR rates than the Low-score group. **(G)** The model’s predictive performance for MPR. NCIT, neoadjuvant chemoimmunotherapy; MPR, major pathological response; AUC, area under the curve; MRD, minimal residual disease; MAE, mean absolute error.

Given the significant association between the status of *NOTCH1* mutation and CIS in baseline tissue samples, as well as P3 MRD status, and pathological response, we performed Lasso regression analysis to identify the most robust predictive factors. This analysis identified both baseline *NOTCH1* mutation status and P3 MRD status as optimal predictors (**Figures 5A, B**). The prediction score for estimating the MPR rate was calculated using the following formula: Score = 19.6326542096909 + (2.89037175789617) × *NOTCH1* + (-21.4244136789189) × P3 MRD, where the presence of a *NOTCH1* mutation or MRD-positive status was assigned a value of 1, and their absence was assigned 0. A higher score indicates a greater probability of achieving MPR. The optimal classification threshold, determined using the Youden index, was 21.07784009. A nomogram was subsequently constructed based on the logistic regression model to facilitate individualized prediction of patient response to NCIT (**Figure 5C**). The model demonstrated excellent goodness-of-fit and calibration, as evidenced by a Brier score of 0.067, a MAE of 0.038, and a *p*-value of 0.972 via the Hosmer-Lemeshow test (**Figure 5D**). To rigorously evaluate the risk of overfitting due to the small sample size, internal validation via 1,000 bootstrap resamples was performed, yielding a bias-corrected calibration slope of 0.970 and an intercept of -0.224 (**Figure 5D**), which collectively indicated high consistency between the predicted and observed probabilities. ROC curve analysis demonstrated significantly superior discriminative capability of the prediction score AUC = 0.954, 95% CI: 0.880-1.000) compared to both *NOTCH1* mutation status (AUC = 0.769; 95% CI: 0.617-0.922) and P3 MRD status (AUC = 0.882; 95% CI: 0.778-0.986) individually (**Figure 5E**). To further ensure the robustness of our findings, LOOCV was employed as a stringent internal validation measure. Notably, the LOOCV-AUC remained robust at 0.866 (**Supplementary Figure S4**), confirming that the model’s predictive accuracy was stable and not driven by individual outliers. Subsequently, patients were stratified into Low-score and High-score groups based on the optimal threshold (21.07784009). The High-score group exhibited a significantly higher MPR rate than the Low-score group (*p* < 0.0001; **Figure 5F**). Finally, the model’s predictive performance for MPR was evaluated, demonstrating a sensitivity of 85.7%, specificity of 94.1%, PPV of 85.7%, and NPV of 94.1% (**Figure 5G**).

## Discussion

Although the incidence of ESCC in China is declining, most patients are still diagnosed at locally advanced or advanced stages, posing significant clinical challenges (17). Neoadjuvant chemoimmunotherapy followed by surgery is now a standard treatment, supported by clinical trials (3, 5, 18). In our cohort, the pCR and MPR rates after neoadjuvant chemotherapy combined with PD-1 inhibitors were 13.8% and 31.0%.

Predictive biomarkers for neoadjuvant therapy response are well established in non-small cell lung cancer (NSCLC), such as PD-L1 expression and TMB (19). However, our findings and data from the TD-NICE trial (18) confirmed these are not reliable predictors in ESCC, highlighting disease-specific differences. ITH is associated with reduced efficacy of clinical treatments. Cui et al. found that CIN may drive ITH at all molecular levels, and ITH in ESCC is characterized by multiple independent mechanisms (20). In our study, we observed that MPR group exhibited significantly higher CIN but lower ITH, although this difference was not statistically significant.

Resistance to therapy remains common, with up to 40% of locally advanced ESCC patients not benefitting from neoadjuvant regimens, partly due to underlying genetic alterations (21). Research indicates that aberrant activation of the NOTCH signaling pathway is heavily linked to the initiation, progression, invasion, and metastasis of various tumors (22). A study on neoadjuvant chemotherapy resistance in esophageal cancer found a significantly higher detection rate of *NOTCH1* missense mutations in patients who did not respond to neoadjuvant chemotherapy (23). The RATIONALE-302 trial demonstrated that a *NOTCH1* mutation could serve as a predictive biomarker for extended overall survival (OS) in patients treated with tislelizumab versus chemotherapy (18.4 months compared to 5.3 months) (5). Additionally, the presence of *NOTCH1* mutations was linked to an enhancement of type I interferon (IFN-I) signatures and a decrease in B cells and neutrophils, indicating that NOTCH1 deficiency may foster a more immunologically active tumor microenvironment, thereby boosting the effectiveness of anti-PD-1 therapy (24). Our study found a higher *NOTCH1* mutation rate in the MPR group, which aligns with previous studies suggesting these mutations could enhance immunotherapy effectiveness, possibly by altering tumor immune microenvironment activity. Further studies are warranted to clarify the mechanisms and therapeutic potential of targeting *NOTCH1* in ESCC.

Avoiding radical esophagectomy can markedly improve postoperative quality of life, making accurate identification of pCR after neoadjuvant therapy crucial. While imaging and machine learning approaches show some promise (25), MRD monitoring based on ctDNA is emerging as a robust noninvasive method for assessing treatment efficacy and prognosis (8, 26). In our study, all patients had detectable ctDNA before therapy. Post-treatment, 45.8% became MRD-negative, and importantly, all patients who achieved MPR were MRD-negative, while most non-MPR cases remained MRD-positive. MRD negativity post-therapy strongly correlated with favorable pathological response, showing 100% sensitivity and NPV, confirming MRD status as a valuable marker for therapy efficacy. This was further reinforced by our analysis of quantitative ctDNA dynamics, which revealed that the magnitude of ctDNA clearance (both maxVAF and concentration) relative to baseline at P3 was significantly greater in MPR patients.

However, there was no correlation between plasma MRD status on C2D1 and pathological response. Moreover, baseline-to-P2 changes (percent decrease and log2 FC) in maxVAF and ctDNA concentration also did not significantly differ between MPR and non-MPR groups, suggesting that MRD cannot reliably predict the final outcome of NCIT during the early stage of treatment (after one cycle). Although both MPR and non-MPR groups demonstrated early reductions in ctDNA levels, at treatment completion only MPR patients achieved full clearance, whereas over 50% of non-MPR patients showed increased ctDNA concentration or maxVAF relative to C2D1. These results suggest that although most ESCC patients achieved a favorable molecular response (indicating reduced tumor burden) after the first cycle of NCIT, a subset experiences disease progression or develops resistance during subsequent treatment. This lack of response manifests molecularly as either MRD conversion to positivity or an increase in ctDNA levels relative to C2D1. These data indicate that MRD testing is most informative after completion of neoadjuvant therapy, not after just one treatment cycle.

To enhance prediction of MPR, we developed a simplified model integrating baseline *NOTCH1* mutation and post-NCIT MRD status. This model achieved robust performance, outperforming each biomarker alone, and effectively distinguished patients likely to achieve favorable pathological outcomes. Such approaches may enable more tailored treatment strategies and help identify candidates for organ-preserving management.

Despite these encouraging results, this study is limited by its small sample size and short postoperative follow-up, restricting the assessment of long-term prognostic value. The limited number of total cases (n=29) and MPR events (n = 9) may reduce statistical power and may result in optimistic estimates of effect sizes and model performance; therefore, the predictive model should be interpreted as preliminary. Larger, independent cohorts are needed to externally validate our findings, particularly the predictive model for MPR. Additionally, further research should address optimal MRD assessment timing and its utility in guiding surgical decisions.

In conclusion, this study demonstrated the clinical significance of dynamic MRD monitoring in predicting pathological response to NCIT in locally advanced ESCC patients, with the post-treatment window optimal for assessment. Integrating baseline *NOTCH1* mutations with post-NCIT MRD status significantly improved MPR prediction performance. These findings may have potential value for optimizing NCIT strategies in such patients.

## Data Availability

The original contributions presented in the study are included in the article/**Supplementary Material**, further inquiries can be directed to the corresponding author/s.

## Supplementary material

Supplementary Figure S1 Somatic genomic alterations identified by targeted next-generation sequencing in 29 ESCC patients.

Supplementary Figure S2 Comparison of ctDNA-detected genomic alteration frequencies between MPR and non-MPR groups, using Fisher’s exact test to determine statistical significance.

Supplementary Figure S3 Quantitative ctDNA dynamics from baseline (P1) to on-treatment (P2) and post-treatment (P3) stratified by pathological response.

Supplementary Figure S4 Internal validation of the predictive model using leave-one-out cross-validation (LOOCV).

Supplementary Table S1 Detailed molecular spectrum and predicted functional impacts of *NOTCH1* mutations detected in the baseline tumor samples.

Supplementary Table S2 Comparison of TMB, CIS and ITH between MPR and non-MPR groups.
