# Supplementary figures and images for: Genomic alterations and dynamic molecular residual disease monitoring predict pathological response to neoadjuvant chemoimmunotherapy in esophageal squamous cell carcinoma
Source: Front Immunol. 2026 Apr 30;17:1681959. doi: 10.3389/fimmu.2026.1681959 (PMC13171771; doi:10.3389/fimmu.2026.1681959)

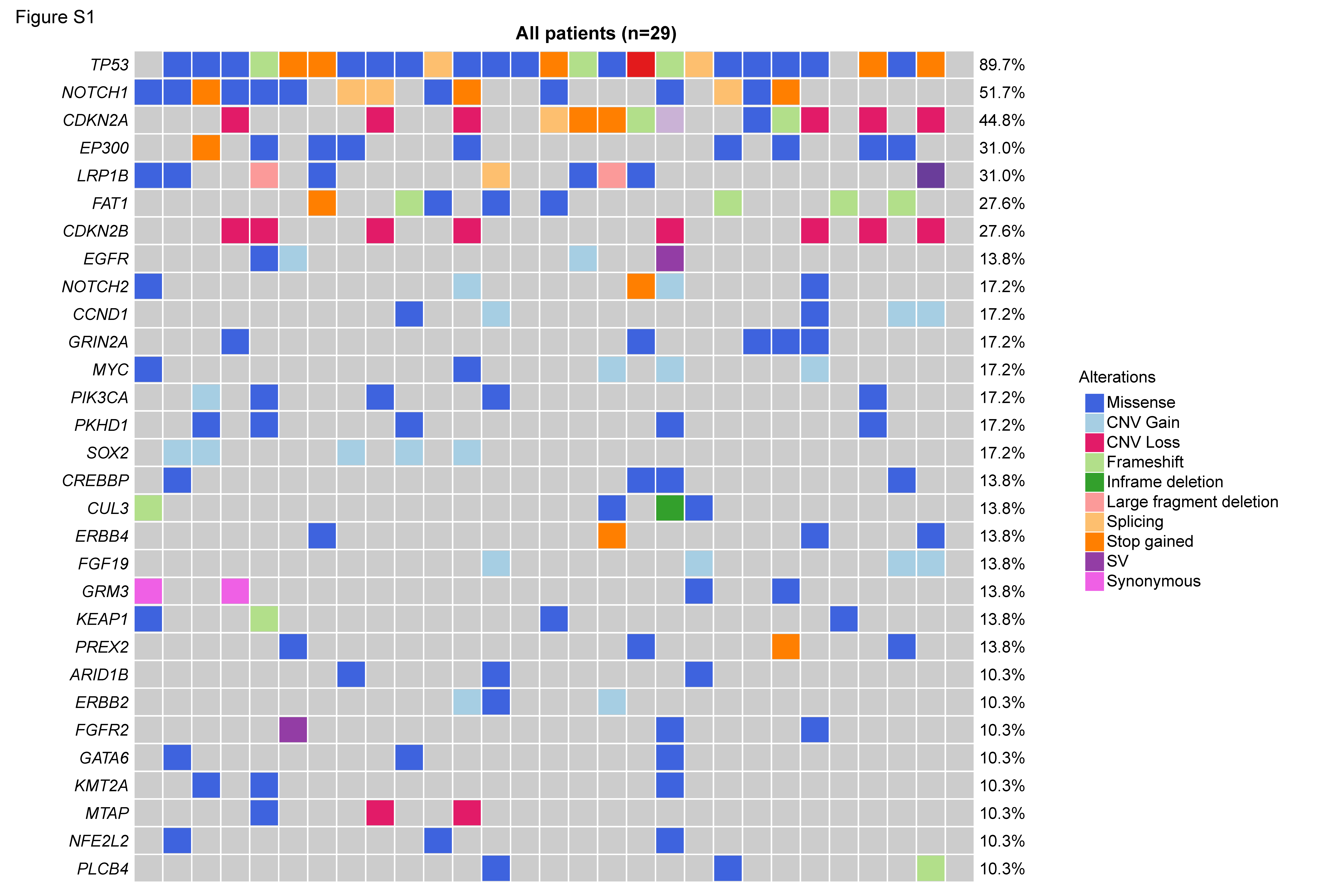

Supplement: Supplementary Figure S1 — Somatic genomic alterations identified by targeted next-generation sequencing in 29 ESCC patients. [file Image1.tif]

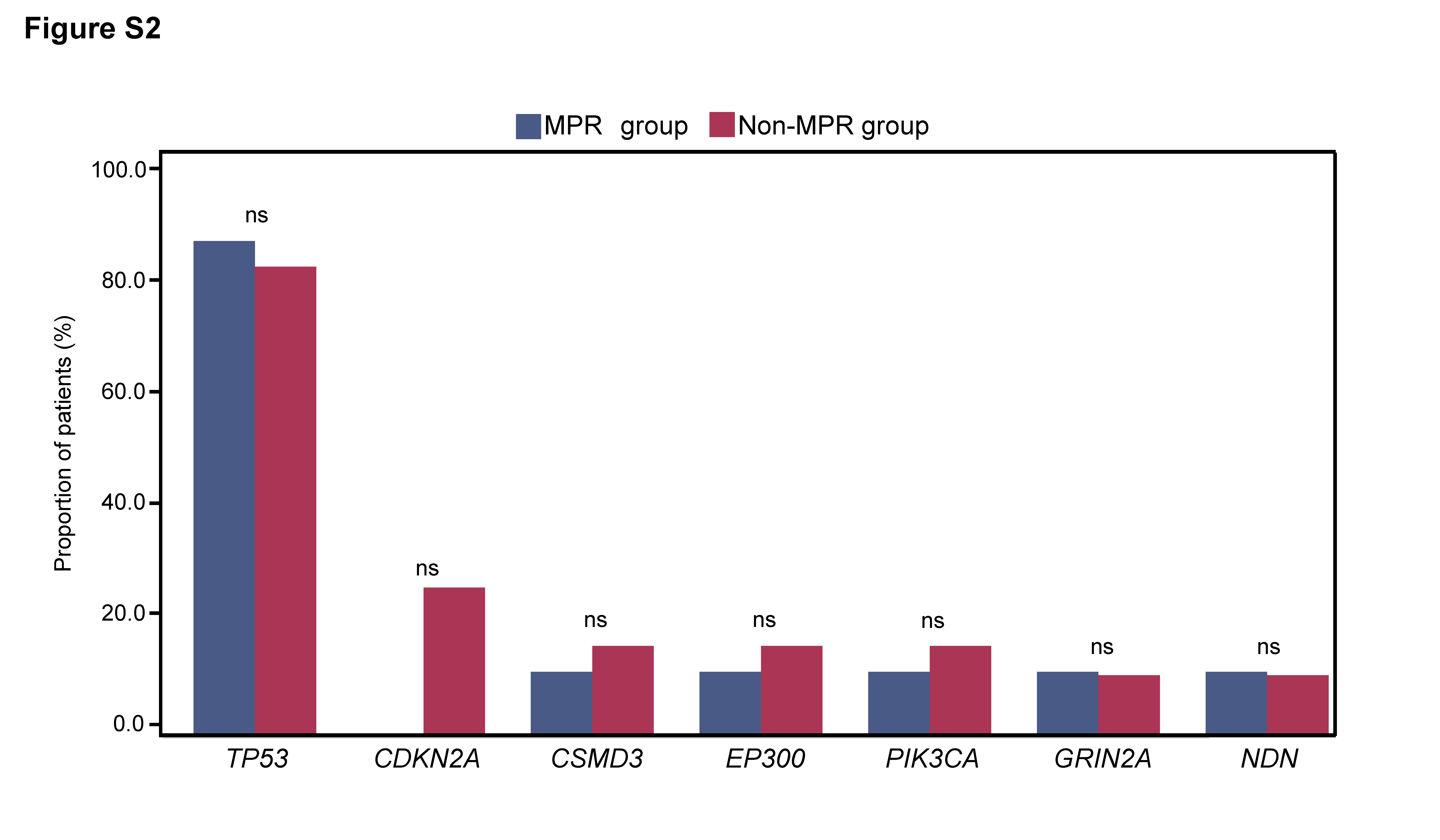

Supplement: Supplementary Figure S2 — Comparison of ctDNA-detected genomic alteration frequencies between MPR and non-MPR groups, using Fisher’s exact test to determine statistical significance. [file Image2.tif]

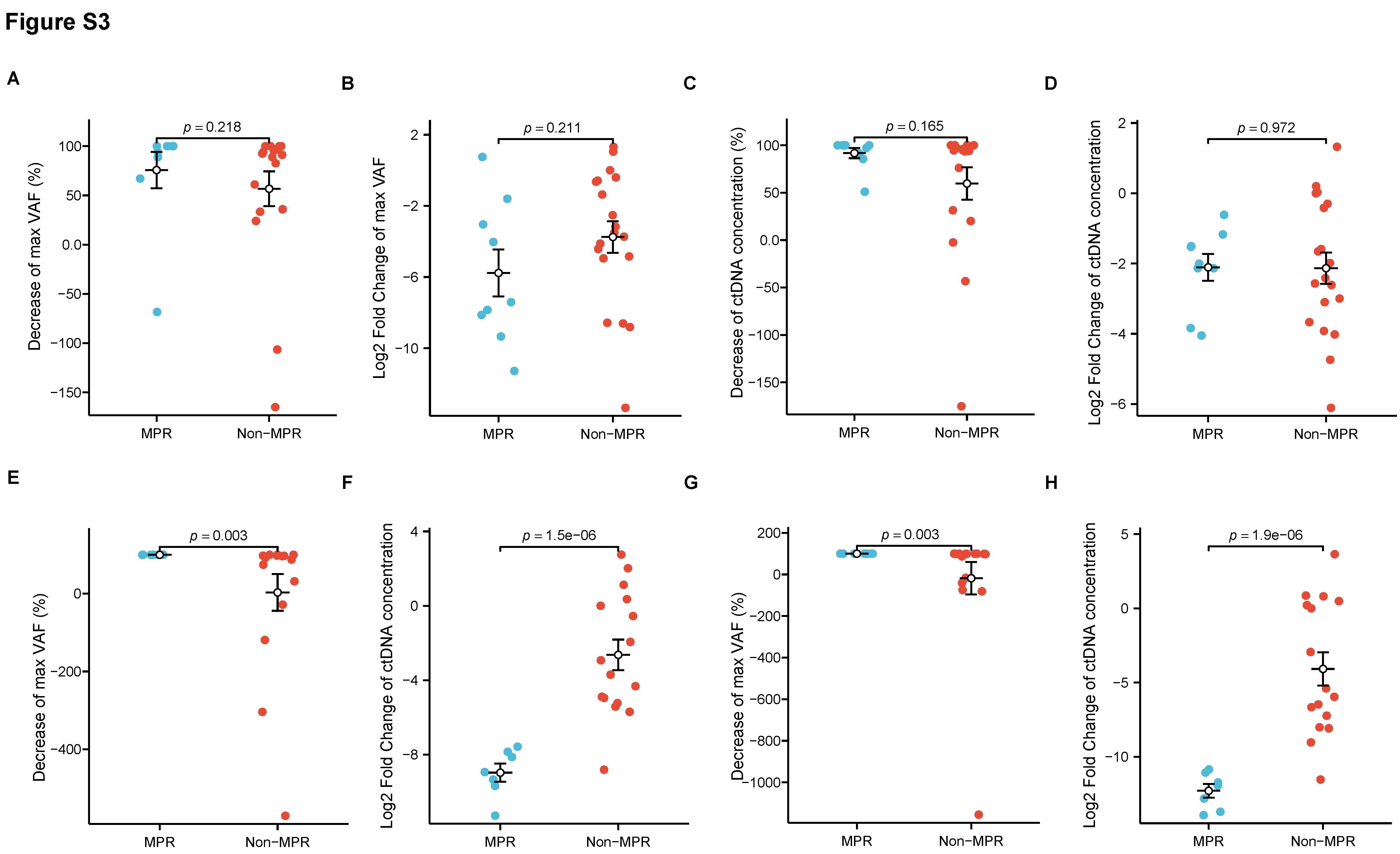

Supplement: Supplementary Figure S3 — Quantitative ctDNA dynamics from baseline (P1) to on-treatment (P2) and post-treatment (P3) stratified by pathological response. [file Image3.tif]

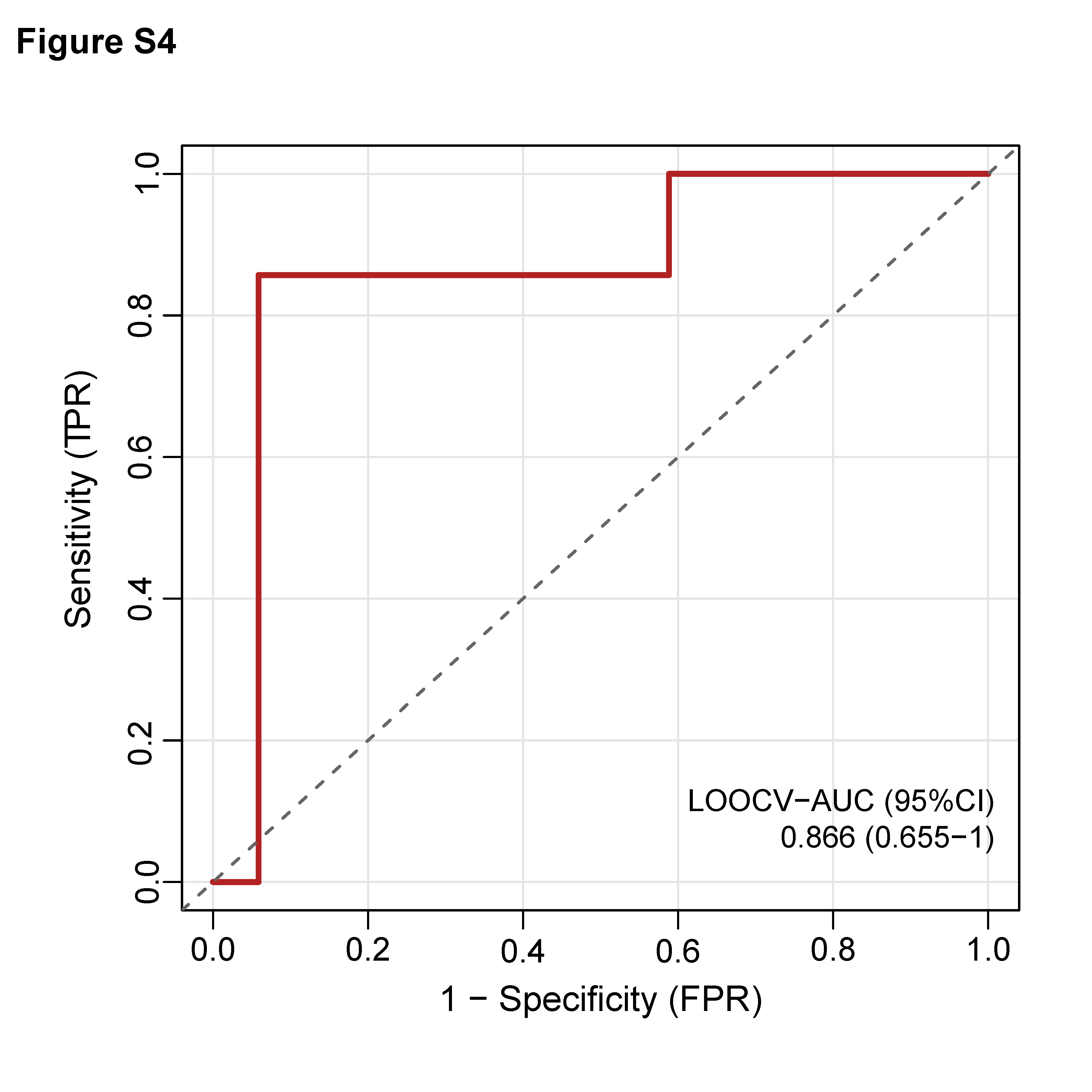

Supplement: Supplementary Figure S4 — Internal validation of the predictive model using leave-one-out cross-validation (LOOCV). [file Image4.tif]
